# Damage Severity Assessment of Multi-Layer Complex Structures Based on a Damage Information Extraction Method with Ladder Feature Mining

**DOI:** 10.3390/s24092950

**Published:** 2024-05-06

**Authors:** Jiajie Tu, Jiajia Yan, Xiaojin Ji, Qijian Liu, Xinlin Qing

**Affiliations:** School of Aerospace Engineering, Xiamen University, Xiamen 361102, China; jiajietu@stu.xmu.edu.cn (J.T.); yanjiajia@xmu.edu.cn (J.Y.); xiaojinji@stu.xmu.edu.cn (X.J.); qijianliu@xmu.edu.cn (Q.L.)

**Keywords:** multi-layer complex structure, damage severity assessment, damage information extraction, ladder feature mining

## Abstract

Multi-layer complex structures are widely used in large-scale engineering structures because of their diverse combinations of properties and excellent overall performance. However, multi-layer complex structures are prone to interlaminar debonding damage during use. Therefore, it is necessary to monitor debonding damage in engineering applications to determine structural integrity. In this paper, a damage information extraction method with ladder feature mining for Lamb waves is proposed. The method is able to optimize and screen effective damage information through ladder-type damage extraction. It is suitable for evaluating the severity of debonding damage in aluminum-foamed silicone rubber, a novel multi-layer complex structure. The proposed method contains ladder feature mining stages of damage information selection and damage feature fusion, realizing a multi-level damage information extraction process from coarse to fine. The results show that the accuracy of damage severity assessment by the damage information extraction method with ladder feature mining is improved by more than 5% compared to other methods. The effectiveness and accuracy of the method in assessing the damage severity of multi-layer complex structures are demonstrated, providing a new perspective and solution for damage monitoring of multi-layer complex structures.

## 1. Introduction

Multi-layer complex structures consist of multiple layers of metallic, non-metallic or composite laminates. They are components formed through the integration of special processes such as structural coating, gluing and bonding [1]. The structures can overcome the limitations of single material performance, with a variety of combination forms and excellent comprehensive performance. In addition, the structures have the advantages of high design efficiency, good corrosion and wear resistances, so they are increasingly used in large equipment engineering structures [2]. For example, solid rocket engines [3], aluminum-plastic composite pipes [4], steel-plastic composite pipes [5] and external sound-absorbing tiles of submarines [6] all use multi-layer complex structures.

However, during service, multi-layer complex structures are prone to different forms of damage because of static loads, external impacts, fatigue and other factors [7]. Among different damages, debonding damage is difficult to detect due to its smallness, concealment and complexity. However, debonding damage, as one of the most vulnerable types of damage in multi-layer complex structures, not only affecting the strength and stability of the structure but also leading to the loss of the overall function of the structure, resulting in significant economic losses and accidents. Therefore, timely detection and assessment of the debonding damage severity are of paramount significance in engineering to prevent sudden damage and structural failure [8].

Debonding damage in multi-layer complex structures is usually located in hidden areas, making it difficult to detect by conventional non-destructive testing (NDT) methods. In addition, long-term online monitoring of structures during service by NDT is very difficult, especially for space-constrained or large-size multi-layer complex structures [9]. Therefore, structural health monitoring (SHM) technology is applied in this study as an effective monitoring method for multi-layer complex structures. By deploying the network of sensors on the structure, SHM can acquire the structural status in real time and realize the intelligent monitoring of latent and extended structural damage [10].

There are many monitoring methods for SHM, such as the ultrasonic guided wave method [11], the strain measurement method [12], the electromechanical impedance method [13] and the self-sensing method [14]. Currently, the ultrasonic guided wave (Lamb wave) monitoring method based on piezoelectric transducers (PZT) is one of the most promising methods for damage monitoring [15]. It is capable of monitoring large areas and has the advantages of fast response, sensitivity to small damages, high identification accuracy and cost-effectiveness, which make it suitable for health monitoring of complex plate structure [16].

In recent years, many researchers have started to focus on damage information extraction methods, such as feature fusion, feature mapping or data fusion. The damage information extraction methods can obtain more effective feature sets or datasets to characterize the features, thereby improving the effectiveness of SHM. For instance, Tang et al., combined optimal path extraction with principal component analysis (PCA) techniques to improve damage recognition accuracy through feature fusion [17]. Song et al., proposed a feature fusion method based on multi-head attention mechanisms, which successfully realized the cross-fertilization of local and global features [18]. Wang et al., proposed an algorithm-centered domain-based adaptive feature mapping method for enhancing guided wave information capable of characterizing damage [19]. Liao et al., introduced a method for damage information enhancement and multi-path data fusion, which performed well in damage localization and size quantification [20]. Furthermore, with the booming field of machine learning, techniques such as Convolutional Neural Networks (CNN) [21], Support Vector Machines (SVM) [22] and Random Forest (RF) [23] have been widely used in SHM. However, all of the above damage information extraction methods have been used to assess the damage of a single material or the same type of layup material. Some researchers have also conducted studies on laminated structures made of different materials. Lugovtsova et al., analyzed the propagation of guided waves in multi-layer complex plate of aluminum and carbon fiber-reinforced composite (CFRP) by using the scaled-boundary finite-element method (SBFEM) [24]. This study contributes to the understanding of wave propagation laws in multi-layer structures. However, since multi-layer complex structures are usually bonded by adhesives, it is difficult to realize high-fidelity simulations, and thus there is a difference between the simulation results and experimental results. As a result, many researchers have begun to focus on how to perform damage monitoring directly through experimental signals and signal analysis. Yang et al., built an online monitoring system for fully automatic detection using a coordinate transformation method and an ellipse location algorithm in a hydrogen storage vessel (multi-layer complex structure) [25]. Lugovtsova et al., estimated the size, depth and location of impact damage in the delaminated aluminum–CFRP composite plate by wavenumber mapping techniques [26]. Mehrabi et al., extracted important indices, such as the energy index (EI) and the amplitude index (AI), from the experimental signals to calculate the length of debonding damage [27]. However, it is difficult for independent features to strongly characterize the degree of damage. The dispersion of damage features of multi-layer complex structures is strong because each layer of material is different. There are few methods applicable to feature processing and damage information extraction to assess the severity of damage in multi-layer complex structures.

The focus of this paper is on aluminum-foamed silicone rubber multi-layer complex structures. The focus is on the assessment method of debonding damage severity applicable to multi-layer complex structures. In this paper, the propagation law of Lamb waves in this structure is analyzed by experimental signals. A damage information extraction method based on ladder feature mining is proposed. The method is able to optimize and screen effective damage information through a ladder-type damage extraction method. It is suitable for evaluating the severity of debonding damage in aluminum-foamed silicone rubber, a novel multi-layer complex structure. This method contains ladder feature mining stages of damage information selection and damage feature fusion. During the stage of damage information selection, a dual localization method that operates in both the time and frequency domains, changing from regional frequencies to precise frequencies, is proposed. During the stage of damage feature fusion, a multi-dimensional dual feature fusion method, transitioning from high-dimensional to low-dimensional, is proposed. Using the damage information extraction method based on ladder feature mining, we can effectively locate the monitoring frequency band that is most sensitive to damage, improve the recognition accuracy of damage severity and optimize the monitoring effect.

The layout of this paper is as follows. Section 2 introduces the theory of Lamb wave propagation in plate structures. Section 3 proposes a damage information extraction method based on ladder feature mining. Section 4 describes the experimental setup and arrangement. Section 5 presents the results and discussion. Finally, Section 6 summarizes the conclusions.

## 2. Lamb Wave Propagation Theory in Plate Structures

**Figure 1:**
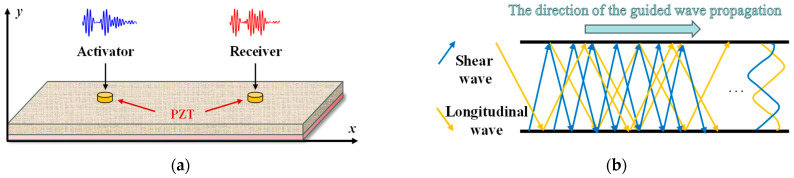
Lamb wave propagation. (**a**) Schematic diagram of Lamb wave excitation–reception. (**b**) Formation of Lamb in a free plate.

The Lamb wave is an ultrasonic guided wave that propagates in thin plates with similar thickness and wavelength. As ultrasonic waves propagate through a thin plate, shear and longitudinal waves couple with each other to form a unique type of stress wave: the Lamb wave [28]. The process of Lamb wave generation and propagation, shown in Figure 1, involves multiple reflections between discontinuous interfaces of sound waves in the medium, which interfere with each other to form complex waveforms and geometric dispersion [29]. 

**Figure 2:**
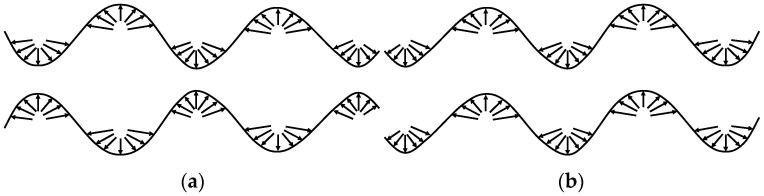
Lamb wave modes. (**a**) S mode; (**b**) A mode.

The Lamb wave has dispersive and multimodal properties. It can be categorized into symmetric and antisymmetric Lamb waves, according to structural properties, as shown in Figure 2. In the symmetric mode (symmetric wave, S mode), the vibration direction of the plasmas is consistent with the propagation direction of the Lamb wave, which is mainly affected by the radial displacement of the particles in the plane. In the anti-symmetric mode (anti-symmetric wave, A mode), the vibration direction of the plasmas is perpendicular to the propagation direction of the Lamb wave, which is mainly dominated by the normal displacement of the particles out of the plane. Both modes contain multilevel components that propagate independently in the plate and have different propagation characteristics [30].

The S and A modes are represented by Rayleigh–Lamb equations [30]:

(1) $$\frac{\tan(\frac{\beta h}{2})}{\tan(\frac{\alpha h}{2})}=-\frac{4k^{2}\alpha \beta }{{\left(\beta^{2}-k^{2}\right)}^{2}}$$

(2) $$\frac{\tan(\frac{\beta h}{2})}{\tan(\frac{\alpha h}{2})}=-\frac{{\left(\beta^{2}-k^{2}\right)}^{2}}{4k^{2}\alpha \beta }$$

(3) $$\alpha^{2}=(\frac{\omega^{2}}{C_{L}^{2}})-k^{2},\beta^{2}=(\frac{\omega^{2}}{C_{T}^{2}})-k^{2}$$

Equations (1) and (2) represent the S mode and the A mode, respectively, where *k* is the wave number in the horizontal direction, *h* is the thickness of the plate, *ω* is the angular frequency, *C_L_* and *C_T_* are the longitudinal and shear wave velocities, respectively. The wave equation determines the multi-mode and frequency dispersion properties of the Lamb wave.

In the quantitative monitoring of structural damage, the group velocity is usually used to calculate the propagation time or propagation distance of the Lamb wave. The group velocity refers to the lamb wave packet propagation velocity, which is the key to damage monitoring. The group velocity, *Cg*, is usually defined as follows:

(4) $$C_{g}=\frac{\partial \omega }{\partial k}$$

As the Lamb wave propagates through the structure to the damage site, one part of the wave transmits and continues to propagate forward, while the other part generates a reflected wave at the damage site. Since the damage may lead to changes in the medium’s morphology, such as changes in the symmetry of the plate structure in the thickness direction, it can exacerbate the dispersion phenomenon and lead to rapid attenuation of the signal energy, among other things. Therefore, the amplitude, phase and other characteristic parameters of the Lamb wave signal may change. The dispersion curve and characteristic parameters of the Lamb wave can be used to obtain the wave propagation time or distance to further determine the location or extent of damage.

## 3. Method

**Figure 3:**
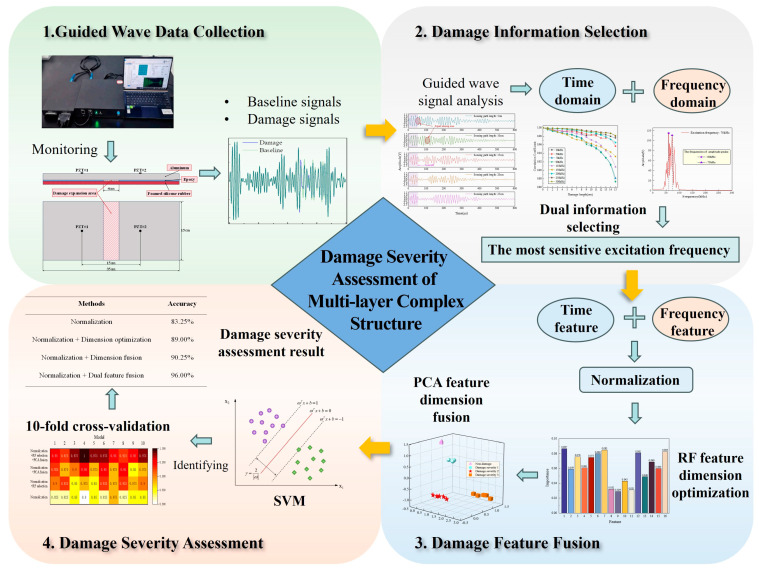
The flowchart of the experiment.

The flow of damage severity assessment of multi-layer complex structures based on a damage information extraction method with ladder feature mining is shown in Figure 3. It includes four parts: guided wave signal collection, damage information selection, damage feature fusion and damage severity assessment.

The proposed damage information extraction method based on ladder feature mining is mainly divided into a damage information selection stage and a damage feature fusion stage. The methods of the two stages are highlighted below.

### 3.1. Damage Information Selection Stage

In the damage information selection stage, a dual localization method from the regional frequency to the precise frequency is proposed. The frequency information that best characterizes the damage is jointly mined by the synergistic work of time domain and frequency domain analyses. The specific steps are as follows.

1. According to the propagation law and propagation characteristics of the Lamb wave in the multi-layer complex structure, a suitable monitoring frequency range A_0_–A_1_ is selected.
2. When the multi-layer complex structure is damaged to different degrees, we compare the damage signals with the baseline signals under the monitoring frequency range A_0_–A_1_. The time domain feature information is analyzed after extracting the time domain features, such as the correlation coefficient, maximum value, variance and root mean square. The frequency selection range is gradually narrowed down, and, finally, a most sensitive frequency band B_0_–B_1_ is determined. In this experiment, the selected optimal frequency band difference is 20 kHz.
3. Within the monitoring frequency of B_0_–B_1_, the frequency domain analysis based on Fast Fourier Transform (FFT) is carried out on the scattered signals of different degrees. Through the peak analysis of the spectrum, a common sensitive frequency C_0_ is obtained, which is the optimal damage monitoring frequency.

The proposed dual information selecting method in both time and frequency domains achieves the localization of excitation signals transitioning from regional frequencies to precise frequencies of the multi-layer complex structure. The most sensitive frequency bands to damage are selected, laying a solid foundation for the subsequent feature fusion stage.

### 3.2. Damage Feature Fusion Stage

In the damage feature fusion stage, a multi-dimensional dual feature fusion method, transitioning from high-dimensional to low-dimensional, is proposed. Multidimensional signal features that can characterize the damage of multi-layer complex structures are taken as the research object. The feature dimensions are optimized by the cross-dimensional dual feature fusion method. The mining of potential principal component information is performed to achieve the purpose of feature approximate reduction and damage information enhancement.

#### 3.2.1. Multi-Dimensional Feature Extraction

Due to the strong dispersion of damage features in multi-layer complex structures, multiple features that can characterize the damage information are first extracted from different angles during the damage feature fusion stage. The features extracted based on the time domain are shown in Table 1. The features include the correlation coefficient, the maximum, the peak-to-peak, the root mean square and the variance of the signals. The generation of damage is often accompanied by changes in waveform amplitude and phase. Therefore, a Hilbert spectral envelope transform is performed on the sensing signals. The peaks of the first and second wave packets and the corresponding time of flight are used as features. The time domain energy ratio coefficients (SDT and SST) and the energy of the Hilbert spectral envelope of the scattered signal are extracted as time domain features. The above time domain features carry different damage information, respectively. The core characteristics of the signal, such as similarity, peak, energy, phase and stability are comprehensively portrayed in several aspects.

In Table 1, *f*_1_(*t*) and *f*_2_(*t*) are the baseline and damage signals, respectively. *H_n_^f^*^(*t*)^ is the *n*th wave packet of the Hilbert spectral transform of the *f*(*t*) signal. *N* is the total number of samples. *N_max_* is the number of samples at which the peak point is located.

The features of the frequency domain based on the FFT are shown in Table 2. The peak is a direct reflection of the intensity of a particular frequency component. The energy reflects the distribution of the signal in the frequency domain. The frequency domain energy ratio coefficients, SDS and SSS, are also important frequency domain features that provide additional information for signal analysis. In Table 2, *F^f^*^(*t*)^ is the frequency domain signal obtained after the FFT of the *f*(*t*) signal.

#### 3.2.2. Dual Feature Fusion

In the dual damage feature fusion stage, in order to ensure that each feature has a more balanced impact on the model, a normalization method is used in the preprocessing stage. Normalization can solve the problem of features with different magnitudes and large differences in the range of values. It makes the range of values between different features consistent, thus optimizing the performance of the model. The formula for normalization is the following [31]:

(21) $$X_{NEW(i)}^{\left(j\right)}=\frac{X_{i}-X_{\min}}{X_{\max}-X_{\min}}$$

The feature dimension is fused for the first time by the RF algorithm. RF is an integrated learning algorithm whose base unit is a decision tree model [32]. The RF algorithm has the function of evaluating the importance of features by calculating the degree of contribution of different features to each decision tree model [33]. This contribution can be represented by calculating the mean square error (MSE) of out-of-bag (OOB) data.

The MSE is added as a performance measure to find the optimal value of the decision tree. On the basis of this decision tree, the importance of each feature is measured by calculating the average contribution of features. Then, feature selection is performed based on feature importance to complete feature dimension optimization. The steps are as follows.

1. Obtain the value of the best decision tree. The feature matrix *X_NEW_* and the target variable matrix *y* are separated. Different numbers of decision trees are iterated by setting the seed of the random number generator. Each run evaluates the MSE and selects the number of decision trees *N_t_* that minimizes the MSE. The formula is as follows:

(22) $$meanResult=\frac{1}{numRuns}\sum_{i=1}^{numRuns}MSE(Model.pre(X_{NEW}),y)$$

(23) $$N_{t}=\arg\min_{numRuns}meanResult$$

*Model.pre*(*X_NEW_*) is the model predicting the input data *X_NEW_*, and *y* is the true target variable. The purpose of Equation (22) is to calculate the MSE of the model in each run (*numRuns* times) and then take the average. Equation (23) represents finding *N_t_* that minimizes the *meanResult* by traversing different *N_t_* values.

2. Obtain feature importance. Construct the RF model using the optimal number of decision trees. The feature importance scores are obtained by enabling the calculation of feature importance for out-of-bag (OOB) sample prediction errors. The importance of features φ can be expressed as follows:

(24) $$\varphi^{\left(j\right)}=\frac{1}{N_{t}}\sum_{i=1}^{N_{t}}\left(MSE_{OOB2i}-MSE_{OOBi}\right)$$

*MSE_OOB_*_2*i*_ is the mean square error of the prediction of the *i*th tree using the OOB data after adding random noise. *MSE_OOBi_* is the mean square error of the prediction of the *i*th tree using the original OOB data.

3. Feature selection: The features are ranked according to their importance. The top 50% of features in terms of importance are filtered from highest to lowest to complete the first damage feature fusion. The feature set obtained from this feature dimension optimization process is *X_NEW_*’.

The feature dimension is fused for the second time by PCA. PCA is an information fusion technique that preserves data information and performs dimensional fusion. For the selected feature matrix *X_NEW_*’, the covariance matrix is obtained by calculating the transpose matrix of the feature matrix. Then, principal components are obtained by eigenvalue decomposition. The dataset after PCA fusion is *X*’. The equation is the following [34]:

(25) $$X^{\prime }={X_{NEW}}^{\prime }\cdot V$$

where *V* is the matrix containing the principal components. The fusion of data dimensions is achieved by selecting the number of principal components to be retained.

In the damage feature fusion stage, a dual feature fusion method of feature dimension optimization and feature dimension fusion is proposed. The method realizes the multi-dimensional fusion process from high to low dimensions. It is suitable for damage monitoring of multi-layer complex structures with strong damage feature dispersion. The RF feature dimension optimization method and the PCA feature dimension fusion method are mentioned at this stage. The two methods have their respective advantages and limitations. However, the dual feature fusion method, which combines the two methods, is able to overcome their respective limitations and has the advantages of strong model generalization, low risk of overfitting, applicability to small sample data and low time cost. The comparison of these methods is shown in Table 3.

## 4. Experimental Program

### 4.1. Multi-Layer Complex Structure Preparation

**Figure 4:**
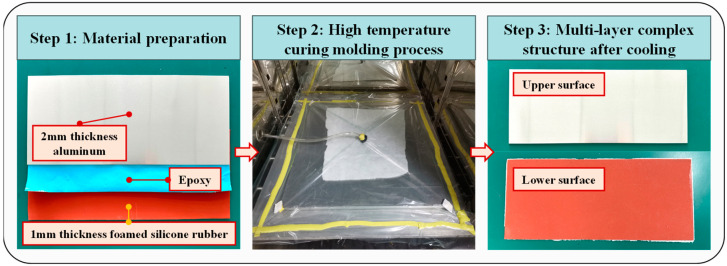
Multi-layer complex material preparation process.

The multi-layer complex structure in this experiment consists of a 350 mm × 150 mm × 2 mm 6061 aluminum plate and a 350 mm × 150 mm × 1 mm foamed silicone rubber plate. The bonding material for the laminated structure is GXA120 toughened epoxy film. It offers excellent stability in joining multi-layer laminated structures to the core material. The preparation process of the experimental material is shown in Figure 4.

First, the surface of the plates was cleaned and dried to remove impurities and moisture. Then, the treated sheets and films were stacked on the bottom mold in the specified order and covered with the top mold. The mold was sealed with a vacuum bag to achieve a uniform force on the material during the curing process. It was placed in a high temperature oven and cured at 105 °C for 180 min. Finally, the cured multi-layer complex structure was annealed and removed. The prepared aluminum-foamed silicone rubber multi-layer complex structure was obtained.

### 4.2. Sensor Signal Test

**Figure 5:**
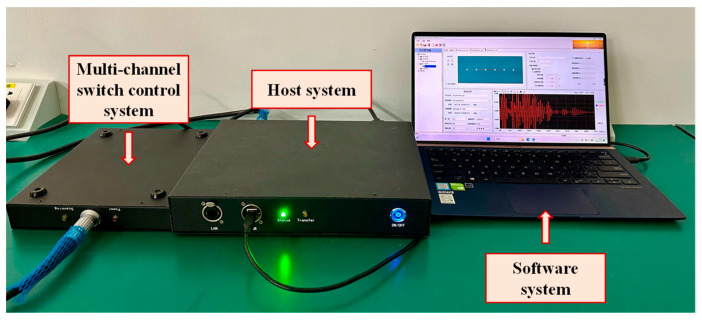
Multi-channel damage monitoring system.

The damage monitoring system is shown in Figure 5. The monitoring system comprises a 128-channel switch control system, a host system, a software system and associated accessories. The dimensions of the switch control system are 223 mm × 201 mm × 49 mm, and the host system measures 300 mm × 226 mm × 50 mm. The monitoring system was developed by Dalian Junsheng Technology Co. It allows signal collection by providing a five-cycle Hanning window sinusoidal waveform on the transmitter while obtaining measurements on the receiver. The main technical indicators of the equipment are shown in Table 4.

A five-cycle sine wave was used as the excitation signal in the experiment. PZT sensors of 8 mm diameter and 0.33 mm thickness were used as both the activator and the receiver. An epoxy adhesive (Hysol EA 9394) was used to firmly adhere the PZT sensors to the structure. This adhesive can be cured at room temperature and has a high bond strength. 

**Figure 6:**
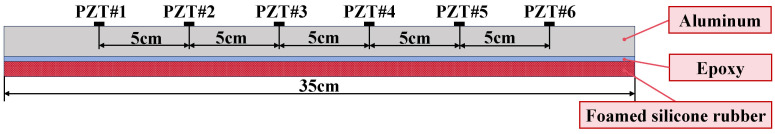
The sensor network layout of the distance test.

Before the damage monitoring experiments were conducted, the multi-layer complex structure was tested for optimal sensing distance. As shown in Figure 6, six PZT sensors were linearly adhered to the surface of the aluminum plate at 5 cm intervals. This produced sensing signals at distances of 5 cm, 10 cm, 15 cm, 20 cm and 25 cm. After analyzing and comparing the sensing signals at different distances, the optimal sensing distance can be determined.

Repeatability tests were also performed on the sensing signals of the multi-layer complex structure. The repeatability test was performed at the optimal sensing distance. Baseline signals were captured in the frequency range of 20–300 kHz. Subsequently, experimental signals were recaptured after 1, 3, 5, 10 and 15 days. The acquired signals were analyzed for correlation with the initial baseline signal. The correlation index (*C_i_*) is expressed in Equation (26). *C_i_* represents the correlation between the signals on the *i*th day and signals on the 0th day; *f*^0^(*t*) denotes the sensing signal in the initial state (on the 0th day) under the excitation signal frequency of *m* kHz; *f^i^*(*t*) represents the sensing signal on the *i*th day under the excitation signal frequency of *m* kHz.

(26) $$C_{i}=\frac{\sum_{m=20}^{300}corr(f^{0}(t),f^{i}(t))}{281}$$

### 4.3. Verification of Damage Monitoring Ability

**Figure 7:**
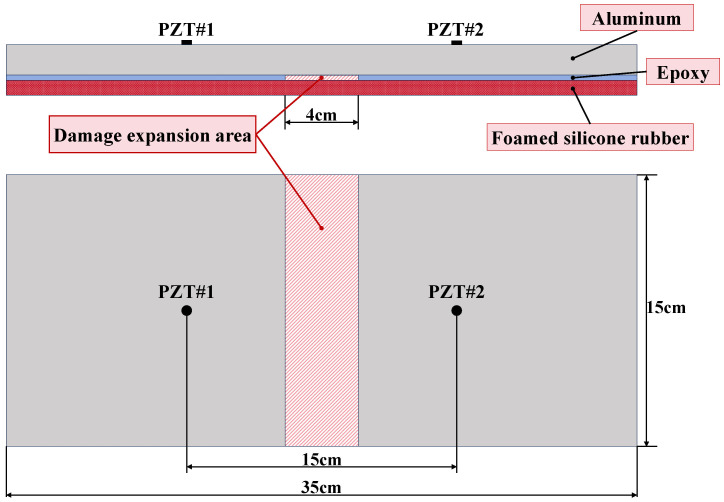
Structural damage dimensions and location diagram.

The debonding of the structure can occur due to stress concentrations or adhesive failure. The damage may also cause partial damage to the bonding interface material during damage extension. Therefore, we continuously used a knife to remove the epoxy resin film to simulate the debonding extension process without using Teflon or other paper materials to simulate the damage. The width of the damage for the experiment was set at 4 cm. Damage length was increased from 0 to 15 cm at 1 cm intervals, as shown in Figure 7. In the assessment of damage severity, the damage length of 1–5 cm is classified as damage severity 1, the damage length of 6–10 cm is classified as damage severity 2 and the damage length of 11–15 cm is classified as damage severity 3.

During damage monitoring, two PZT sensors were fixed on the material surface at a distance of 15 cm (the optimal monitoring distance). One of them was used to transmit the signal and the other was used to receive the signal. The excitation signal was a 5-period tone burst signal (modulated by a Hanning window). The signal gain was 25 dB, the amplitude of the excitation signal was 100 V, and the sampling rate was 12 MHz/s. The excitation frequency range was set to 20–300 kHz by analyzing the Lamb wave signals in the structure.

## 5. Results and Discussion

### 5.1. Guided Wave Signal Analysis of Multi-Layer Complex Structure

#### 5.1.1. Structural Signal Testing and Analysis

**Figure 8:**
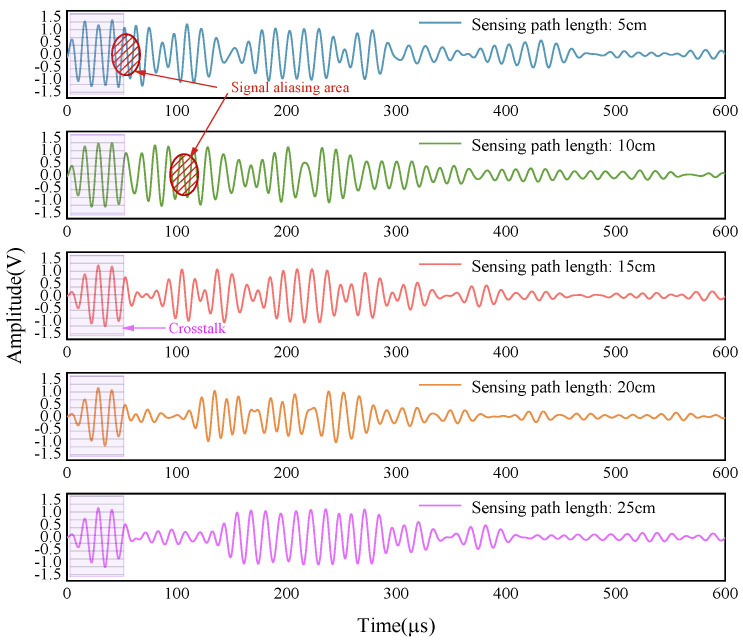
Comparison of the received signals of the multi-layer complex structure at different sensing distances.

The distance test results of the multi-layer complex structure are shown in Figure 8. When the sensing distance is 5 cm or 10 cm, the five-wave peak signal received for the first time is easily mixed with other modal signals and crosstalk signals, which makes subsequent signal analysis difficult. At a sensing distance of 25 cm, the signal amplitude is relatively small. The proximity of the sensor to the edge of the structure produces a boundary reflection signal that may affect the accuracy of the direct signal. In contrast, at sensing distances of 15 cm and 20 cm, the signal quality is better, especially at 15 cm, where the first five-wave peak signal is more complete, facilitating signal processing and analysis. Therefore, the sensing distance of 15 cm is the optimal monitoring distance.

The results of the repeatability experiments of the sensing signals are shown in Table 5. Specifically, the correlation index *C_i_* of the signal on day 1 was 99.87%. The *C_i_* values of day 3, 5, 10 and 15 are 98.62%, 97.97%, 96.45% and 96.40%, respectively. The values of all *C_i_* exceed 95% within 15 days, further confirming the stability and reproducibility of the sensor materials and signals.

#### 5.1.2. Damage Signal Processing

**Figure 9:**
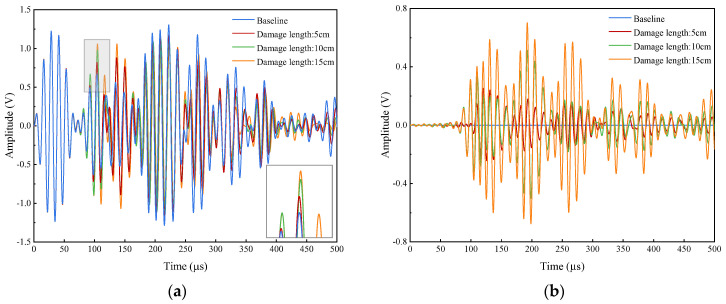
Comparison of signals without damage and signals with different damage lengths. (**a**) Baseline signals and damage signals. (**b**) Undamaged and damaged scattered signals.

The baseline signal and the damage signals for damage lengths of 5 cm, 10 cm and 15 cm, respectively, are shown in Figure 9a. As can be seen from the partially enlarged detail in the lower right corner, the peak of the direct wave gradually increases, and the phase gradually shifts to the right as the damage length increases. All damage signals are converted into the corresponding scattered signals shown in Figure 9b.

**Figure 10:**
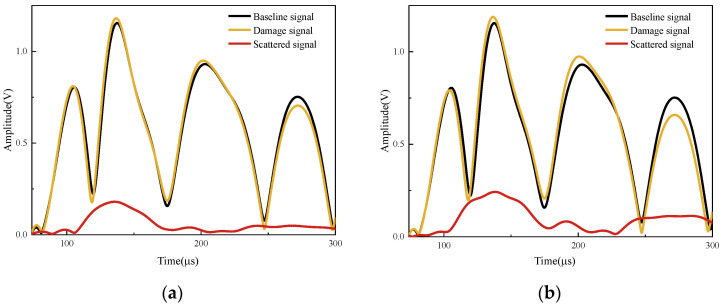

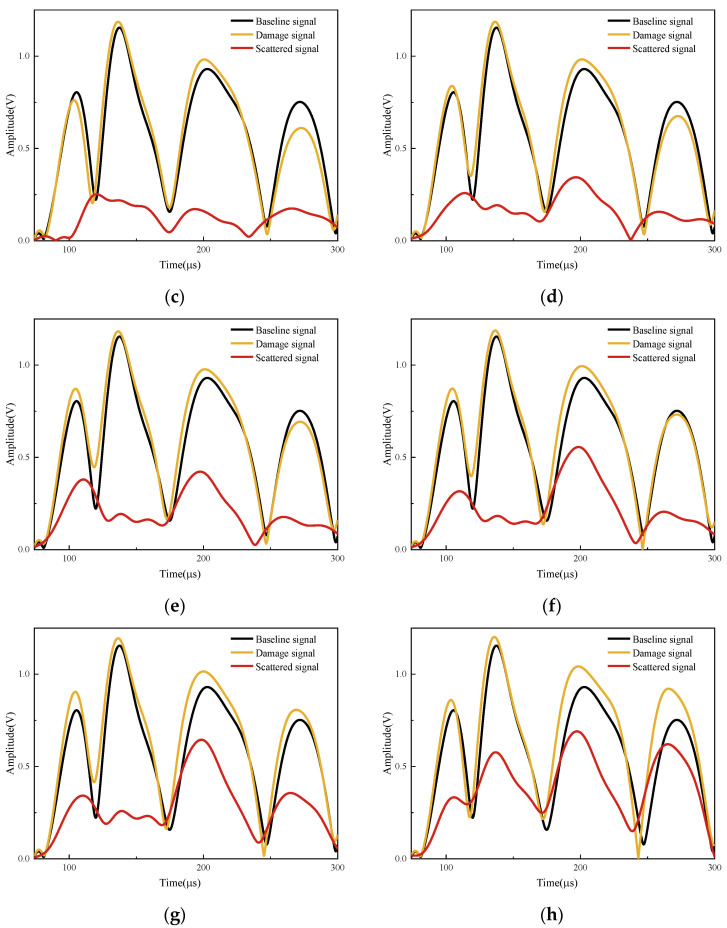
Hilbert transform of baseline, damage and scattered signals of different damage lengths. (**a**) Damage length: 1 cm. (**b**) Damage length: 3 cm. (**c**) Damage length: 5 cm. (**d**) Damage length: 7 cm. (**e**) Damage length: 9 cm. (**f**) Damage length: 11 cm. (**g**) Damage length: 13 cm. (**h**) Damage length: 15 cm.

In the Hilbert transform, the sampling time window is 70–300 μs, which avoids crosstalk signals in the early stage, reflected waves in the late stage and multimodal waves. Figure 10 shows the variation process of damage signal and scattered signal when the damage lengths are 1 cm, 3 cm, 5 cm, 7 cm, 9 cm, 11 cm, 13 cm and 15 cm, with an excitation frequency of 75 kHz. Overall, the first three wave packets are more sensitive to damage occurrence. The amplitude of the scattered signal increases gradually with the increase in the damage length. When the damage occurs in the early stage, the damage is mainly reflected in the change of the second wave packet. However, as the damage gradually extends from the edge to the sensor’s connection line, the sensitivities of both the first direct wave packet and the third wave packet to the damage gradually increase. Therefore, subsequent damage features will be performed for the first three wave packets.

### 5.2. Dual Damage Information Selection Based on Time and Frequency Domains

**Figure 11:**
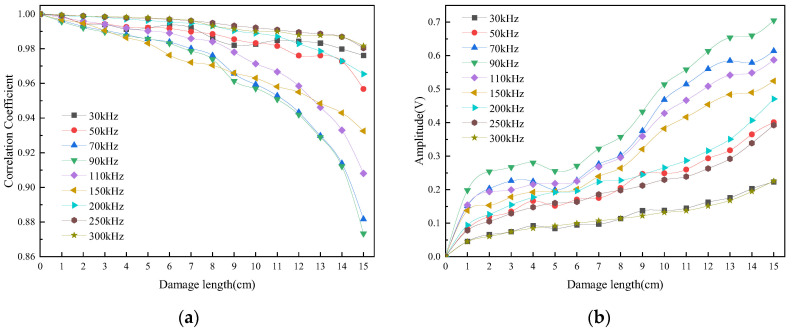
The correlation coefficient of the acquired signal and the maximum of the scattered signal at the excitation frequency of 30–300 kHz. (**a**) Correlation coefficient. (**b**) Maximum.

This section describes the damage information selection method based on a combination of time and frequency domains. The correlation between the damage signal and the baseline signal in the excitation frequency range of 20–300 kHz is explored in Figure 11a. The shapes of the correlation coefficient curves are different for different frequencies. However, the general trend is that the correlation coefficient decreases with increasing damage length. The most significant change in the correlation coefficient occurs in the 70–90 kHz band. It indicates that the sensitivity of damage monitoring is higher in this frequency band. Similarly, the maximum values of the scattered signal are shown in Figure 11b. It shows a non-linear relationship with the damage length, but the most significant changes are observed in the 70–90 kHz band. Thus, this step localizes the frequency from a large range of 20–300 kHz to a small range of 70–90 kHz. It is the first step in the process of damage information selection based on the time domain analysis.

**Figure 12:**
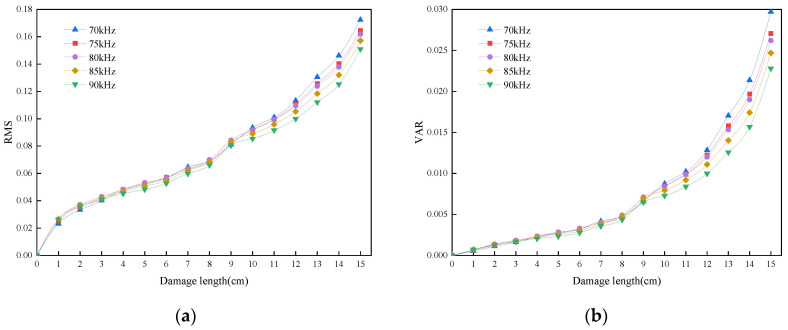
The RMS and VAR of the scattered signal at the excitation frequency of 70–90 kHz. (**a**) Root mean square. (**b**) Variance.

From the point of view of signal energy, the root mean square and variance reflect the overall energy level of the scattered and acquired signals. Figure 12 shows the signals in the frequency range of 70–90 kHz. It can be noticed that the root mean square shows a similar linear growth trend with increasing damage length, while the variance shows a similar exponential growth, indicating that the energy-centered analysis is also an important characterization parameter. In addition, the curves of root mean square and variance in the frequency range of 70–90 kHz show small differences at different frequencies, respectively. It has been proven that the energy characterizations are similar for different frequencies in this band. Further selection of damage information through the frequency domain is needed.

**Figure 13:**
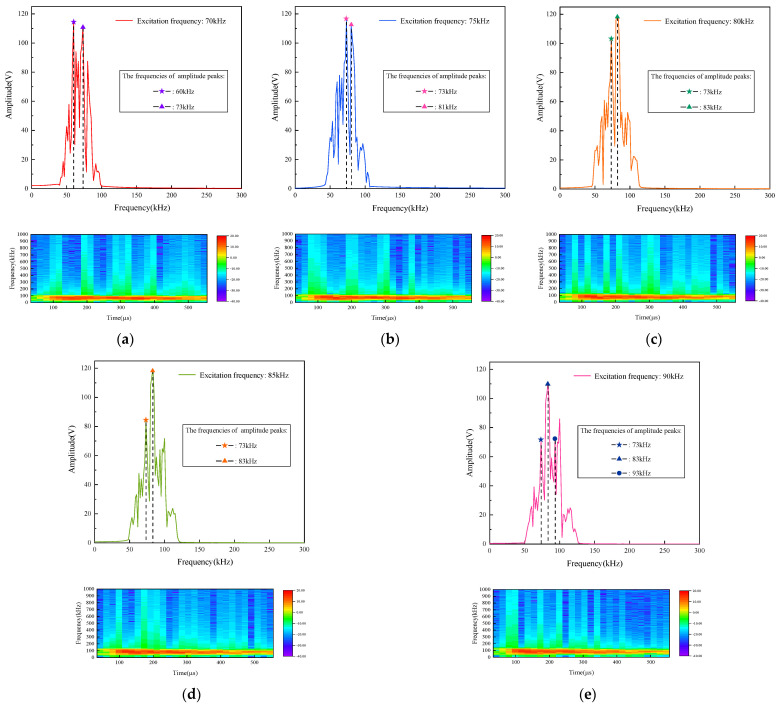
Spectrum and time frequency diagrams of scattered signals: (**a**) 70 kHz; (**b**) 75 kHz; (**c**) 80 kHz; (**d**) 85 kHz; (**e**) 90 kHz.

After performing FFT on signals with different damage lengths at an excitation frequency of 70–90 kHz, it can be found that most of the spectral peaks occur at 73 kHz. Figure 13 shows the spectrogram plots after FFT and the time-frequency plots after Short Time Fourier Transform (STFT) for the damage length of 5 cm. It can be clearly seen from the figure that a peak at 73 kHz appears in all the spectrograms from 70 to 90 kHz, which fully indicates that the 73 kHz frequency is the most sensitive to damage. Meanwhile, the time frequency diagram also demonstrates the variation of the damage signal in time and frequency. Therefore, in the subsequent feature fusion and damage quantization analysis, we use 73 kHz as the excitation frequency for feature extraction of multi-layer complex structures. Therefore, this step localizes the frequency from a small range of 70–90 kHz to a precise frequency of 73 kHz. The second step of damage information selection based on the frequency domain analysis is realized.

### 5.3. Dual Damage Feature Fusion Based on Dimension Optimization and Dimension Fusion

**Figure 14:**
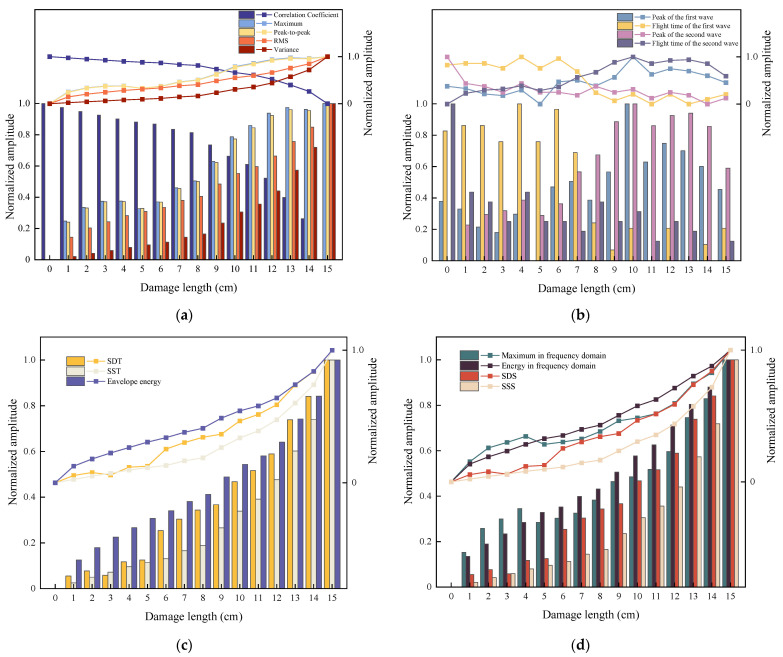
Normalized features. (**a**) Features 1–5. (**b**) Features 6–9. (**c**) Features 10–12. (**d**) Features 13–16.

After dual damage information selection based on time and frequency domains, the experiment locates the exact excitation frequency information of 73 kHz. On this basis, this section will further feature mine the damage information. A total of 16 damage features that can characterize the damage signal are extracted according to Table 1 and Table 2. Meanwhile, all the features are normalized, and the normalized features are shown in Figure 14.

**Figure 15:**
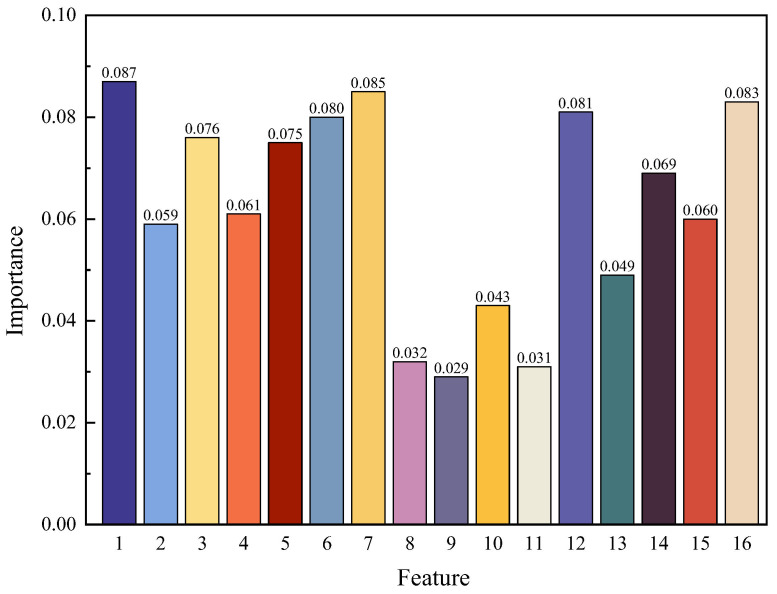
Importance of individual features.

After feature normalization, the importance of each feature is evaluated using the RF algorithm. The output of each decision tree is traversed, compared and analyzed to produce the most robust decision. Figure 15 illustrates the importance of the 16 features. After the analysis, it can be seen that eight features (1, 3, 5, 6, 7, 12, 14 and 16) are more important and are selected. Therefore, the step completes the dimension optimization from 16 to 8 features, which is the first step in damage feature fusion.

**Figure 16:**
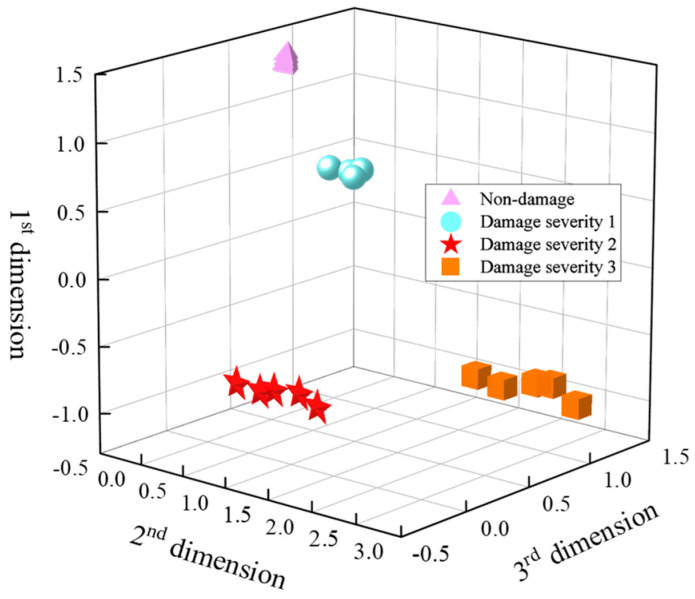
Different damage severities based on dual feature fusion.

The damage is classified into three categories: damage severity 1, damage severity 2 and damage severity 3. The optimized dimensions are fused using PCA. The fusion results are shown in Figure 16. This method is effective in distinguishing the damage severity, which proves that the features can effectively characterize damage severity after dimension optimization and dimension fusion. Therefore, the step completes the information fusion of 8-dimensional features, which is the second step in damage feature fusion.

### 5.4. Damage Severity Identification Results

A SVM classifier is used to evaluate the damage degree to verify the effectiveness of the proposed damage information extraction method based on ladder feature mining. Testing sets are randomly selected for validation from the undamaged samples and from the samples with three different damage severity degrees, respectively. Ten-fold cross-validation is used to validate the results to reduce the chance of the accuracy results. There are ten model sample selections. Among them, the sample selection for Model 1 is shown in Table 6.

**Figure 17:**
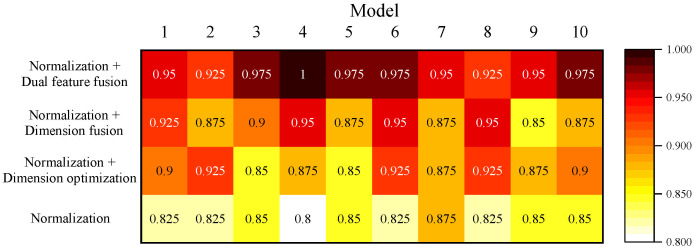
The damage severity identification results of different methods by ten iterations of ten-fold cross-validation.

The damage severity identification accuracies of the normalization, normalization + dimension optimization, normalization + dimension fusion and normalization + dual feature fusion methods are compared sequentially. Figure 17 shows the accuracies of the different methods after ten-fold cross-validation. Table 7 provides the final identification accuracies. It is clear that the highest identification accuracy of 96% is achieved by the dual feature fusion method. This method improves the accuracy by 12.25% compared to the accuracy without feature fusion, and it improves accuracy by 7% and 5.75% compared to the two methods that performed only one fusion. Both methods that perform feature fusion only once have higher identification accuracy rates compared to the methods without feature fusion. It confirms that the proposed method can accurately and efficiently recognize damage severity. In addition, feature fusion processes of feature dimension optimization and feature dimension fusion both contribute to identification accuracy.

## 6. Conclusions

In this article, the propagation of guided wave signals in aluminum-foamed silicone rubber multi-layer complex structures and the processing of damage information are analyzed. A damage information extraction method with ladder feature mining is proposed. During the damage signal selection stage of the method, a dual localization method in both time and frequency domains, transitioning from regional frequencies to precise frequencies, is proposed. A precise frequency of 73 kHz is localized from a large regional frequency range of 20–300 kHz to select the most sensitive excitation frequency for damage detection. During the signal fusion stage of the method, a multi-dimensional dual feature fusion method from high to low dimensions is proposed. It achieves RF-based feature dimension optimization and PCA-based feature dimension fusion. The results show that the proposed damage information extraction method is effective for damage severity assessment. The accuracy of damage severity identification using this method reaches 96%, which is improved by 12.25% compared to other methods. However, there are still some shortcomings. The sample size of real damage obtained in this study is small, and the sensor layout is sparse. The accuracy of localization and quantification needs to be further explored. In addition, the damage extension path is relatively simple, but its generalization ability needs to be improved.

## Figures and Tables

**Table 1:** Time domain feature parameters.

Feature Number	Parameter	Calculation Equation	
1	Correlation coefficient	$Corr=\frac{Cov(f_{1}(t),\;f_{2}(t))}{\sigma_{f_{1}(t)}\sigma_{f_{2}(t)}}$	(5)
2	Maximum value	$Maximum=\max(f_{1}(t)-f_{2}(t))$	(6)
3	Peak-to-peak value	$V_{pp}=\max(f_{1}(t)-f_{2}(t))-\min(f_{1}(t)-f_{2}(t))$	(7)
4	Root mean square	$RMS=\sqrt{\frac{1}{T}\int {\left(f_{1}(t)-f_{2}(t)\right)}^{2}dt}$	(8)
5	Variance	$VAR=\frac{1}{T}\int (f_{1}(t)-f_{2}(t)-\frac{1}{T}\int \left(f_{1}(t)-f_{2}(t)\right)dt{)}^{2}dt$	(9)
6	Peak of the first wave	$P_{1}=\max(H_{1}^{f_{2}(t)})$	(10)
7	Flight time of the first wave	$FT_{1}=\frac{N_{\max(H_{1}^{f_{2}(t)})}}{N}T$	(11)
8	Peak of the second wave	$P_{2}=\max(H_{2}^{f_{2}(t)})$	(12)
9	Flight time of the second wave	$FT_{2}=\frac{N_{\max(H_{2}^{f_{2}(t)})}}{N}T$	(13)
10	SDT	$SDT=\frac{\int {\left(f_{2}(t)\right)}^{2}dt-\int {\left(f_{1}(t)\right)}^{2}dt}{\int {\left(f_{1}(t)\right)}^{2}dt}$	(14)
11	SST	$SST=\frac{\int {\left(f_{2}(t)-f_{1}(t)\right)}^{2}dt}{\int {\left(f_{2}(t)\right)}^{2}dt}$	(15)
12	Envelope energy	$Energy_{Envelope}=\int H^{f_{1}(t)-f_{2}(t)}dt$	(16)

**Table 2:** Frequency domain feature parameters.

Feature Number	Parameter	Calculation Equation	
13	Maximum value	$Maximum_{F}=\max(F^{f_{1}(t)-f_{2}(t)})$	(17)
14	Energy	$Energy_{F}=\int F^{f_{1}(t)-f_{2}(t)}dF$	(18)
15	SDS	$SDS=\frac{\int {\left(F^{f_{2}(t)}\right)}^{2}dF-\int {\left(F^{f_{1}(t)}\right)}^{2}dF}{\int {\left(F^{f_{1}(t)}\right)}^{2}dF}$	(19)
16	SSS	$SSS=\frac{\int {\left(F^{f_{2}(t)-f_{1}(t)}\right)}^{2}dF}{\int {\left(F^{f_{2}(t)}\right)}^{2}dF}$	(20)

**Table 3:** Comparison of advantages and limitations of different methods.

Method	Advantages	Limitations
RF feature dimension optimization	Robustness Ability to handle non-linear relationships	Easily overfitted Large time cost
PCA feature dimension fusion	Computationally efficient Highly interpretable	Easy to lose information Difficulty in capturing nonlinear relationships
RF + PCA dual feature fusion	Strong model generalization ability Low risk of overfitting Applicable to small sample data Low time cost	Some information may be lost

**Table 4:** Monitoring equipment technical indicators.

Technical Parameter	Value
Excitation frequency range	10–1000 kHz
Conversion rates	48 MHz
Output voltage range	Min: ±10 V; Max: ±60 V
Memory	32,000 Samples
Sampling rates	6, 12, 24, 48 MHz/s
Resolution	12-bit
ADC range	±1 V
Adjustment range	10–40 dB, step: 1 dB

**Table 5:** Repeatability of multi-layer complex plate signals based on correlation index.

Date	Day 1	Day 3	Day 5	Day 10	Day 15
Correlation index (*C_i_*)	99.87%	98.62%	97.97%	96.45%	96.40%

**Table 6:** Sample selection order for Model 1 in ten-fold cross-validation.

Model	1	2	3	4	5	6	7	8	9	10
Non-damage	3	1	2	1	4	2	3	2	1	5
Damage severity 1	6	8	10	8	10	7	8	10	6	10
Damage severity 2	14	15	14	12	11	12	13	13	11	15
Damage severity 3	18	19	20	17	20	16	16	19	18	20

**Table 7:** Damage severity identification accuracies of different methods.

Methods	Accuracy
Normalization	83.75%
Normalization + Dimension optimization	89.00%
Normalization + Dimension fusion	90.25%
Normalization + Dual feature fusion	96.00%

## Data Availability

The data used to support the findings of this study are available from the corresponding author upon request.
